# Point-of-care Ultrasound Identification of Tension Hydrothorax in the Emergency Department: A Case Series

**DOI:** 10.5811/cpcem.1584

**Published:** 2023-08-02

**Authors:** Allison Clark, Peyton Lampley, Vu Huy Tran

**Affiliations:** Aventura Hospital and Medical Center, Emergency Medicine Residency Program, Aventura, Florida

**Keywords:** tension hydrothorax, point-of-care ultrasound

## Abstract

**Introduction:**

Tension hydrothorax is an uncommon emergent condition in which hemodynamic instability and respiratory compromise may occur. Emergency physicians may diagnose tension hydrothorax by point-of-care ultrasound.

**Case Series:**

We discuss the key sonographic features assisting in identification. Four patients with history of malignancy who were found to have tension hydrothorax exhibited the following common ultrasound findings: massive, left-sided pleural effusion; complete, compressive atelectasis; and shift of cardiac structures into the right hemithorax, resulting in right-sided probe placement to obtain cardiac views.

**Conclusion:**

This is the first instance to our knowledge of point-of-care ultrasound findings in tension hydrothorax to be described in the literature.

## INTRODUCTION

Tension hydrothorax (TH) is a rare but life-threatening condition caused by a large pleural effusion displacing mediastinal structures including the heart, lungs, and great vessels. This shift raises intrathoracic pressures causing hemodynamic compromise. Due to tension physiology and obstructive shock, patient presentations range from dyspnea and orthopnea to shock and respiratory insufficiency.^1^

In adults the two most common causes of TH are infectious or malignant processes, although many causes have been identified.^2^ The diagnosis is made based on clinical suspicion and can be supported with imaging via chest radiograph (CXR), point-of-care ultrasound (POCUS), or computed tomography (CT). These modalities may show hemithorax opacification or pleural effusion with contralateral mediastinal displacement.

We describe four patients who presented to our emergency department (ED) with TH with specific, unique cardiothoracic ultrasound findings. To our knowledge, this is the first case series of POCUS findings in TH.

## CASE SERIES

### Case 1

A 56-year-old male of Chinese descent without reported past medical history presented to the ED with days of shortness of breath and pleuritic chest pain. He was diagnosed with a large left pleural effusion via outpatient CXR. Initial vital signs were heart rate (HR) of 98 beats per minute (bpm), blood pressure (BP) of 148/83 millimeters mercury (mm Hg), respiratory rate (RR) of 18 respirations per minute (rpm), oxygen saturation (SpO_2_) of 98% on room air (RA), and an oral temperature of 98.6° Fahrenheit (°F). Physical exam showed no distress with decreased breath sounds on left lung auscultation. Laboratory studies were largely within normal limits, including complete blood count, C-reactive protein (CRP), erythrocyte sedimentation rate (ESR), and chemistry panel.

**Image 1:**
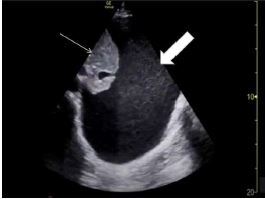
Thoracic point-of-care ultrasound showing atelectatic lung (thin arrow) within a large left pleural effusion (thick arrow), and absent cardiac views from normal left chest windows.

The CXR in the ED showed complete opacification of the left hemithorax with rightward mediastinal shift. Thoracic POCUS showed atelectatic lung within a large left pleural effusion, and absent cardiac views from normal left chest windows (Image 1). Chest CT confirmed a massive left pleural effusion and complete left lung collapse causing rightward mediastinal shift, with concern for a lingular mass. The patient was admitted to the medical service, where an ultrasound-guided thoracentesis removed a total of 3.8 liters (L) of exudative fluid. Pathology of the biopsy from the lingular mass confirmed stage IV adenocarcinoma. The patient was discharged home with a chest tube in situ for close outpatient follow-up.

### Case 2

A 79-year-old female with past medical history of hypertension (HTN), hyperlipidemia, chronic obstructive pulmonary disease on home oxygen therapy, and metastatic lung adenocarcinoma with brain metastases undergoing chemotherapy, presented to the ED with sudden onset of dyspnea. Initial vital signs were as follows: HR 75 bpm; BP 125/80 mm Hg, RR 18 rpm; SpO_2_ 100% on 3L of oxygen via nasal cannula (NC), which was increased from baseline therapy; and an oral temperature of 97.8°F. Her physical exam was significant for mild respiratory distress and bilateral decreased breath sounds. Laboratory studies showed white blood count (WBC) 18.2 ×10^3^/microliters (μL) (reference range 4.0–10.5 ×10^3^/μL), hemoglobin 9.7 grams per deciliter (g/dL) (11.2–15.7 g/dL), D-dimer of 4,632 nanograms per deciliter (ng/mL) (0–316 ng/mL), CRP of 16.5 milligrams/dL (0.0–1.0 mg/dL), and erythrocyte sedimentation rate ESR of 53 millimeters per hour (mm/hr) (0–10 mm/hr). Arterial blood gas showed pH 7.39 (7.35–7.45), partial pressure of carbon dioxide 50 mm Hg (35.0–45.0 mm Hg), and partial pressure of oxygen of 108 mm Hg (80.0–105.0 mm Hg) on 3L NC.

Point-of-care ultrasound revealed a large left pleural effusion with rightward mediastinal shift and a plethoric inferior vena cava with minimal inspiratory collapse. Chest radiograph showed complete left-sided opacification with rightward mediastinal shift. Computed tomography angiography of the chest demonstrated an enlarged left hilar and subcarinal mass when compared to previous CT. New findings included occlusion of the left mainstem bronchus; compression of the mid-esophagus; collapse of the left lung; and a large left pleural effusion. Treatment with thoracentesis was considered but not performed due to the unlikelihood of lung re-expansion and minimal therapeutic benefit. Ultimately, the patient opted for comfort measures only and was discharged to hospice.

**CPC-EM Capsule**

What do we already know about this clinical entity?

*Rare but life threatening, tension hydrothorax occurs when large pleural fluid collections compress and shift mediastinal structures, causing hemodynamic instability.*

What makes this presentation of disease reportable?

*Tension hydrothorax is a rare complication of large pleural effusion that can lead to life-threating tamponade physiology and hemodynamic instability.*

What is the major learning point?

*Adjusting ultrasound probe positioning to the right hemithorax can help decrease time to diagnosis and treatment in emergent scenarios such as tension hydrothorax.*

How might this improve emergency medicine practice?

*If emergency medicine providers understand and can quickly identify tension hydrothorax, it can help prevent further complications or death.*

### Case 3

**Image 2:**
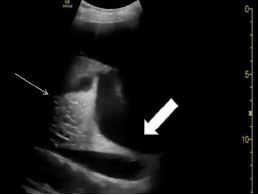
Point-of-care ultrasound in transverse view of the left hemethorax depicting a large, loculated pleural effusion (thick arrow).

A 59-year-old male with history of metastatic neuroendocrine cancer presented to the ED with his hospice nurse for concern of increased dyspnea and decreased oral intake. Initial vital signs were as follows: HR 118 bpm; BP 118/78 mm Hg; RR 20 rpm; SpO_2_ 96% on 4L NC; and temperature of 98.2°F. Physical exam was significant for rales and diminished breath sounds throughout left lung fields. Relevant laboratory studies were as follows: WBC 15.8 ×10^3^/μL (reference range 4.0–10.5 ×10^3^/μL); lactic acid 4.4 millimoles [mmol]/L (0.4–2.0 mmol/L), lactate dehydrogenase of 1,537 units/L (84–246 U/L); CRP 40.59 mg/dL (0–1.0 mg/dL), B-type natriuretic peptide 1,520 units/L (0–217 U/L); troponin I 0.016 ng/mL (0.000–0.034 ng/mL); and D-dimer 812 ng/mL D-dimer units (0–316 ng/mL). Chest radiograph revealed complete opacification of the left hemithorax with rightward mediastinal shift. Point-of-care ultrasound showed large, loculated, left-sided pleural effusion, rightward mediastinal shift, and hyperdynamic left ventricular function on echocardiography (Image 2).

On admission, interventional radiology was consulted for palliative thoracentesis; however, the patient’s healthcare proxy decided against invasive procedures, and he was discharged to hospice.

### Case 4

**Image 3:**
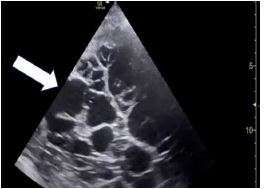
Point-of-care ultrasound in sagittal view of the left hemithorax showing a large, septated pleural effusion (thick arrow) with compressive atelectatic lung (thin arrow).

A 72-year-old female with a history of HTN and lung cancer, undergoing chemotherapy, presented with bilateral lower extremity swelling and dyspnea for one week. Initial vital signs were as follows: HR 108 bpm; BP 170/99 mm Hg; RR 20 rpm; SpO_2_ 95% on RA; and an oral temperature of 98.6°F. Physical exam was significant for a tachypneic, moderately distressed woman with biphasic wheezing and diminished breath sounds bilaterally. Relevant laboratory studies were as follows: platelet level of 132 ×10^3^ U/L (reference range 150–400 × 103 U/L), aspartate aminotransferase of 64 U/L (10–40 U/L), and D-dimer of greater than 5,250 ng/mL (0–316 ng/mL). Chest radiograph showed complete opacification of the left lung and enlarged mediastinum, while POCUS showed a large, left-sided pleural effusion and absent cardiac structures in the left hemithorax (Image 3). The heart was visualized in the right hemithorax showing a small pericardial effusion without evidence of tamponade. Chest CT angiography showed right lower lobe pulmonary embolism, left upper and lower lobe infiltrates, left pleural effusion, and slight rightward midline shift. A bedside thoracentesis removed 3L of straw-colored fluid, improving both respiratory and hemodynamic status, but was complicated by an iatrogenic pneumothorax. She received a tube thoracostomy placed by interventional radiology and was later discharged in stable condition.

## DISCUSSION

A pleural effusion can be considered massive when fluid accumulates to two-thirds or more of the hemithorax. Large volumes of any fluid composition may compress and displace structures within the mediastinum. This can trigger cascading events of decreased venous return, impaired right-sided filling, and diminished cardiac output, all resulting in hemodynamic compromise. At this stage, pathophysiology of TH is comparable to tension pneumothorax. The pleural space is similar in pressure and dynamics as the pericardial space; therefore, build-up in intrapleural pressure results in increased pericardial space pressure. This causes tamponade physiology and similar emergent hemodynamic instability.^3^ For that reason, assessment for tamponade physiology on POCUS is imperative for early intervention and prevention of further complications or death.

The most common causes of pleural effusions are malignancy and infection.^2^ Other potential causes are fluid overload states as seen in heart or liver failure; pulmonary embolism; congenital abnormalities; rheumatologic diseases; trauma; or iatrogenic causes.^2^,^4^ Unusual causes include disseminated endometriosis,^5^ gastric perforation,^4^,^6^ and ventriculopleural shunt.^7^ Most of our cases had known malignancy history, likely having malignant or paramalignant causes of TH.^8^

Patients may present to the ED with normal or abnormal vital signs. Despite its association with shock, TH patients may present with either hypotension or hypertension.^2^ Malignant pleural effusions may arise over long periods, allowing for compensatory mechanisms such as fluid retention and tachycardia.^2^ Physical exam findings may show decreased to absent breath sounds on the affected side, pleural rubs, dullness to percussion^9^ with or without tracheal deviation, and jugular venous distention.^2^

The modalities of physical assessment, radiographs, ultrasounds, and CT imaging can diagnose pleural effusions; each with its advantages and disadvantages. Chest radiographs can show near or complete opacification of the affected hemithorax and mediastinal shift. While quick and cost effective, CXRs require more than 50 mL or 200 mL on upright and supine radiographs, respectively, to detect pleural effusions.^10^ Ultrasound can detect volumes as small as 5 mL but more reliably 20 mL.^10^ For this reason, physical exam findings and plain films could be considered inferior in diagnostic accuracy to ultrasound. Computed tomography can help identify small pleural effusions, assess for complexity such as loculations, and assess for concomitant pleural and parenchymal pathology.^12^

Ultrasound and CT are useful in characterizing, quantifying, and assessing the complexity of the pleural effusion. This information aids in differentiating between transudative vs exudative, supplementary to pleural fluid analysis, and may aid in diagnosing the ultimate cause. Benefits of using POCUS over CT include accessibility, efficiency, reliability, time effectiveness, low cost, safety with lack of ionizing radiation and contrast material, and ability to aid in procedural guidance.^10^

Fortunately for our cohort, our patients initially presented hemodynamically stable. Several showed exam findings we would expect with TH such as dyspnea or hypoxia. All showed hemithorax opacification with mediastinal shift on CXR. Point-of-care ultrasound consistently showed large pleural effusions with atelectatic lung. Two cases had abnormal cardiac ultrasound findings with hyperdynamic function without evidence of tamponade.

All four patients in this cohort exhibited the following POCUS findings: a large, left-sided pleural effusion with compressive atelectasis, with left hemithorax devoid of cardiac views; and rightward displacement of cardiac structures that required repositioning of the transducer to the right parasternal area to obtain adequate views. Additional findings seen in some patients, which would be expected in cases of obstructive shock, are dilation of the inferior vena cava (>2 centimeters) and hyperdynamic echocardiogram findings.

Thus, it is vital to use POCUS to assess for tamponade physiology, as early intervention may prevent further complications or death. Jain et al, who describe a patient with tension hydrothorax due to cirrhosis and portal hypertension, had echocardiogram findings of right atrial and ventricular collapse due to a large transudative pleural effusion. The patient was emergently stabilized with a tube thoracostomy.^9^ Treatment for TH may require urgent vs emergent thoracentesis or tube thoracostomy, dependent on hemodynamic instability. Other cases of TH, regardless of etiology, are treated similarly.^1^,^11^

## CONCLUSION

Clinicians should be cognizant of the following POCUS findings associated with tension hydrothorax: large pleural effusion; left hemithorax devoid of the heart; and rightward cardiac displacement requiring right-sided probe placement in search of possible tamponade physiology. In this scenario, there are additional complications that may change immediate management, such as evaluation for right heart strain, volume status, and cardiac tamponade.

## References

[1] Vinck EE, Garzón JC, Peterson T (2018). Tension hydrothorax: emergency decompression of a pleural cause of cardiac tamponade. Am J Emerg Med.

[2] Demidovich J, Nasseri H, Vasoya A (2012). Tension hydrothorax. Clin Pulm Med.

[3] Chidambaram S, Sangareddi V, Ganesan G (2013). An echocardiographic assessment of cardiovascular hemodynamics in patients with large pleural effusion. Indian Heart J.

[4] Karkhanis VS, Joshi JM (2012). Pleural effusion: diagnosis, treatment, and management. Open Access Emerg Med.

[5] Deal A, Evans D, Counselman FL (2016). Tension hydrothorax from disseminated endometriosis. West J Emerg Med.

[6] Park EY, Rose E (2016). An unusual case of tension hydrothorax in a pediatric patient. J Emerg Med.

[7] Wu TS, Kuroda R (2011). Tension hydrothorax in a pediatric patient with a ventriculopleural shunt. J Emerg Med.

[8] Fenton KN, Richardson JD (1995). Diagnosis and management of malignant pleural effusions. Am J Surg.

[9] Jain S, Erickson HL, Pezzulo A (2016). Ultrasound diagnosis of hemodynamic compromise in a patient with hepatic hydrothorax. Ann Am Thorac Soc.

[10] Soni NJ, Franco R, Velez MI (2015). Ultrasound in the diagnosis and management of pleural effusions. J Hosp Med.

[11] Porter ED, Finley DJ, Phillips JD (2019). Tension hydrothorax secondary to small cell lung cancer. Respirol Case Rep.

[12] Putnam JB (2002). Malignant pleural effusions. Surg Clin North Am.

